# Red blood cell transfusions and the risk of acute respiratory distress syndrome among the critically ill: a cohort study

**DOI:** 10.1186/cc5934

**Published:** 2007-06-06

**Authors:** Marya D Zilberberg, Chureen Carter, Patrick Lefebvre, Monika Raut, Francis Vekeman, Mei Sheng Duh, Andrew F Shorr

**Affiliations:** 1 School of Public Health and Health Sciences, University of Massachusetts, Amherst, P.O. Box 303, Goshen, MA 01032, USA; 2 Ortho Biotech Clinical Affairs, LLC, 430 Route 22 East, Bridgewater, NJ 08807, USA; 3 Groupe d'analyse, 1080 Beaver Hall Hill, Suite 1810, Montreal, Quebec, H2Z 1S8, Canada; 4 Analysis Group, 111 Huntington Avenue, Tenth Floor, Boston, MA 02199, USA; 5 Washington Hospital Center, 110 Irving Street, NW, Washington, DC 20010, USA

## Abstract

**Introduction:**

Recent data indicate that transfusion of packed red blood cells (pRBCs) may increase the risk for the development of acute respiratory distress syndrome (ARDS) in critically ill patients. Uncertainty remains regarding the strength of this relationship.

**Methods:**

To quantify the association between transfusions and intensive care unit (ICU)-onset ARDS, we performed a cohort study within Crit, a multicenter, prospective, observational study of transfusion practice in the ICU which enrolled 4,892 critically ill patients in 284 ICUs in the United States. Diagnostic criteria for ARDS were prospectively defined, and we focused on subjects without ARDS at admission. The development of ARDS in the ICU served as the primary endpoint.

**Results:**

Among the 4,730 patients without ARDS at admission, 246 (5.2%) developed ARDS in the ICU. At baseline, ARDS cases were younger, more likely to be in a surgical ICU, and more likely to be admitted with pneumonia or sepsis than controls without ARDS. Cases also were more likely to have a serum creatinine of greater than 2.0 mg/dl (23% versus 18%) and a serum albumin of less than or equal to 2.3 g/dl (54% versus 30%) and were more severely ill upon ICU admission as measured by either the APACHE II (Acute Physiology and Chronic Health Evaluation II) or SOFA (Sequential Organ Failure Assessment) score (*p *< 0.05 for all). Sixty-seven percent and 42% of cases and controls, respectively, had exposure to pRBC transfusions (*p *< 0.05), and the unadjusted odds ratio (OR) of developing ARDS in transfused patients was 2.74 (95% confidence interval [CI], 2.09 to 3.59; *p *< 0.0001) compared to those never transfused. After age, baseline severity of illness, admitting diagnosis, and process-of-care factors were adjusted for, the independent relationship between pRBC transfusions and ICU-onset ARDS remained significant (adjusted OR, 2.80; 95% CI, 1.90 to 4.12; *p *< 0.0001).

**Conclusion:**

Development of ARDS after ICU admission is common, occurring in approximately 5% of critically ill patients. Transfusion of pRBCs is independently associated with the development of ARDS in the ICU.

## Introduction

Acute respiratory distress syndrome (ARDS) remains a frequent complication of critical illness and is associated with significant morbidity and mortality. Its age-adjusted incidence rate in the United States exceeds 85 cases per 100,000 person-years, and the case fatality rate is near 40% [1]. Illustrating its burden, Rubenfeld and colleagues [1] estimated that ARDS leads to 200,000 intensive care unit (ICU) admissions annually and necessitates 3.6 million inpatient days of care.

Multiple insults can lead to ARDS and range from sepsis to trauma [2-5]. Because the manner in which care is delivered to at-risk patients may contribute to ARDS development, there is a need to better understand process-of-care variables in order to identify those factors associated with an increased risk for ARDS which may be amenable to intervention. Packed red blood cell (pRBC) transfusion represents one specific potential aspect of care in the ICU which may be linked to ARDS. Transfusion-related acute lung injury (TRALI), whose incidence may be 1 in 5,000 pRBC units transfused, has long been recognized as a subtype of ARDS and is generally associated with better outcomes than non-TRALI ARDS [6,7]. Beyond TRALI, though, there is mounting evidence suggesting a nexus between pRBC exposure and ARDS [8-10]. For example, in a study of a conservative transfusion strategy, Hébert and colleagues [8] reported that patients randomly assigned to a higher hemoglobin target were more likely to develop ARDS. Similarly, Gong and colleagues [9], in an analysis of 700 patients, noted that transfusion was significantly associated with the evolution of ARDS. Mechanistically, some speculate that pRBC transfusion could promote ARDS because transfusion activates pro-inflammatory cascades [11,12]. Alternatively, pRBC transfusion alters host defenses, which might predispose to ARDS [13,14].

Given the potential relationship between pRBC use and ARDS, we hypothesized that pRBC transfusions would be independently associated with the development of ARDS. Additionally, we attempted to determine whether there was a dose-response relationship between the two, with greater amounts of transfusion administration associated with an increased incidence of ARDS.

## Materials and methods

### Study overview

This retrospective analysis used data from the Crit study [15]. Crit was a prospective, multicenter, observational study of transfusion practice in ICUs in the United States conducted between August 2000 and April 2001 [15]. A total of 284 ICUs of varying types participated, and the entire study included approximately 5,000 patients. As part of the study, patients had frequent longitudinal assessments of their clinical status and outcomes. Use of pRBCs during and after the ICU stay was also prospectively recorded. Patients were followed until death, hospital discharge, or up to 30 days after ICU admission, whichever occurred first. The institutional review board at each site approved the study, and patients (or their surrogates) provided informed consent. Subsets of this population have since been used to describe transfusion practices in trauma patients [16], as well as in mechanically ventilated patients [17], and to explore the relationship of ventilator-associated pneumonia and bloodstream infections to pRBC transfusions [18,19].

### Subjects and endpoints

Only incident cases of ARDS developing in the ICU were included in the analysis. To ascertain incident cases of ARDS, we excluded from the cohort patients who were admitted to the ICU with a diagnosis of ARDS, as this would not represent a case developing following the exposure of interest (that is, pRBC transfusion). The primary endpoint for this analysis was the development of ARDS defined prospectively based on the North American European Consensus Conference definition of ARDS [20] and relied upon the presence of PaO_2_/FiO_2 _(arterial partial pressure of oxygen/fraction of inspired oxygen) of less than or equal to 200 mm Hg, bilateral infiltrates on frontal chest radiograph, pulmonary artery occlusion pressure of less than or equal to 18 mm Hg when measured, or no clinical evidence of left atrial hypertension. All of the ARDS diagnoses were made prospectively by the investigators in the Crit study. The secondary endpoints examined were ICU and hospital lengths of stay, duration of mechanical ventilation, and ICU and hospital mortality rates, comparing the group developing ARDS (cases) to that not developing ARDS (controls).

### Ascertainment of the blood transfusion exposure

For the ARDS cases, the pRBC transfusions were examined in the time period prior to or at the visit of the first recorded ARDS complication. For the control group, the pRBC transfusions were observed until the end of the study. We used two approaches to categorize individuals with respect to their transfusion exposure: (a) transfusion status formulated as a dichotomous variable (yes/no) and (b) total amount of pRBCs transfused (1 to 2 units, 3 to 4 units, and more than 4 units).

### Identification of potential risk factors

We examined the following potential risk factors for ARDS: patient demographics (age and gender); an admitting diagnosis of pneumonia, sepsis/systemic inflammatory response syndrome (SIRS), neurological disorders, trauma, or post-operative; and ICU type (medical, surgical, or mixed). In addition, we used laboratory data at ICU admission to categorize patients according to the presence or absence of renal failure (defined as serum creatinine of more than 2.0 mg/dl) [9], serum albumin abnormality (more than 2.3 mg/dl or less than or equal to 2.3 mg/dl) [9], and hemoglobin levels. Process-of-care variables were also examined: (a) H_2 _antagonists for ulcer prophylaxis at baseline, (b) treatment with any antibiotic at baseline, and (c) continuous sedation during the observation period, defined as continuous infusion (for at least 24 hours) of any sedative (for example, lorezapam and propofol). For nutritional support, we explored the impact of both parenteral and early enteral nutrition (for example, enteral feeding begun at ICU admission or by ICU day 4) on rates of ARDS.

Two severity-of-illness scores were evaluated during the Crit study: the Acute Physiology and Chronic Health Evaluation II (APACHE II) score and the Sequential Organ Failure Assessment (SOFA) score [21,22]. For the present analysis, to avoid the issue of collinearity by using both severity measures at baseline and to control for the severity of illness while in the ICU, we employed the baseline APACHE II score and a SOFA score indicator, which were generated as follows: (i) for the ARDS group, we used the SOFA score from the visit immediately preceding the visit with the onset of ARDS, and (ii) for the No ARDS group, we used the highest SOFA score observed in the ICU (that is, the worst condition).

### Statistical analysis

A cohort study design was used to examine the independent association between pRBC transfusion exposure and the development of incident ARDS. Univariate descriptive statistics were generated for the ARDS and No ARDS groups. Continuous data were summarized as mean and standard deviation, and the Student *t *test was employed when comparisons of means between the case and control groups were made. Categorical variables were reported as frequency distributions, and chi-square tests were used to test whether the frequency distributions were different between the cases and controls.

To avoid immortal time bias, which generally tends to exaggerate the association between exposure and outcome, multivariate analysis adjusting for time at risk for the outcome event was further conducted to determine independent risk factors for ARDS. Adjusted odds ratios (ORs) of developing ARDS were calculated for the presence of pRBC transfusions, as well as for units of pRBC transfused, by means of stepwise logistic regression analysis to control for covariates. Covariates included in the final regression model were those significant at an alpha level (determined *a priori*) of 10% (that is, *p *value of less than or equal to 0.10) or those with biologic plausibility of relating to ARDS (for example, age, pneumonia, and trauma). The covariates examined were divided into demographic variables, ICU type, admitting diagnosis, severity of illness, comorbid diseases, laboratory data, and ICU processes of care. Because biases may arise from the fact that subjects staying on study for a longer duration of time may have a higher likelihood of developing ARDS, we also incorporated into the model a control variable representing the at-risk time interval between study enrollment and time of first ARDS onset for the cases and between study enrollment and ICU discharge for the controls. Adjusted ORs and their corresponding 95% confidence intervals (CIs) are reported. All tests were two-tailed, and a *p *value of less than 0.05 was predetermined to represent statistical significance. Analyses were carried out using the SAS version 9.1 (SAS Institute Inc., Cary, NC, USA) software package.

## Results

Of the total Crit study population (*n *= 4,892), 408 patients (8.3%) had a diagnosis of ARDS. Of these, 162 with an ARDS diagnosis at admission were excluded from subsequent analyses, resulting in 246 incident ARDS cases (incidence rate, 5.2%). The results of the univariate analysis are presented in Table 1. Although ARDS cases were slightly younger than controls, there was no difference in gender distribution between the two groups. The prevalence of trauma, pneumonia, sepsis/SIRS, and post-operative admitting diagnoses was slightly higher among the patients with ARDS, whereas neurological diagnoses were more common among the control group. ARDS was most prevalent in the combined ICUs, followed by surgical and then medical ICUs. The ARDS group was significantly more likely to have a serum creatinine of more than 2.0 mg/dl, a serum albumin of less than or equal to 2.3 g/dl, a lower baseline hemoglobin, and a significantly greater severity of illness as measured by both APACHE II and SOFA scores when compared to the No ARDS group. Both enteral and parenteral nutrition by ICU day 4 were more likely to be present in the ARDS group than in the control group, as were other process-of-care measures examined (antibiotics and H_2 _antagonists at baseline, as well as continuous sedation).

**Table 1:** Univariate analysis of potential risk factors for ARDS

Variable	No ARDS (*n *= 4,484)		ARDS (*n *= 246)		*P *value	Unadjusted odds ratio
Age in years, mean (SD)	60.3	(18.3)	56.3	(17.3)	0.0008	0.988
Men, number (percentage)	2,468	(55.1)	135	(54.9)	0.9543	0.993
Admitting diagnosis^a^, number (percentage)						
Trauma	578	(12.9)	41	(16.7)	0.0873	1.352
Neurological	760	(17.0)	25	(10.2)	0.0053	0.554
Pneumonia	630	(14.1)	58	(23.6)	< 0.0001	1.887
Sepsis/Systemic inflammatory response syndrome	507	(11.3)	40	(16.3)	0.0180	1.523
Post-operative	892	(19.9)	61	(24.8)	0.0619	1.328
Other	1,741	(38.8)	64	(26.0)	< 0.0001	0.554
ICU type, number (percentage)						
Medical ICU	1,591	(35.5)	74	(30.1)	0.0842	0.782
Surgical ICU	961	(21.4)	81	(32.9)	< 0.0001	1.800
Combined	1,932	(43.1)	91	(37.0)	0.0599	0.776
Nutrition at baseline or ICU day 3–4^a^, number (percentage)						
Enteral nutrition	1,424	(31.8)	116	(47.2)	< 0.0001	1.917
Total parental nutrition	474	(10.6)	67	(27.2)	< 0.0001	3.167
No nutrition or none recorded	2,617	(58.4)	67	(27.4)	< 0.0001	0.267
Process of care, number (percentage)						
Antibiotics at baseline	907	(20.2)	27	(11.0)	0.0004	0.486
H_2 _antagonist at baseline	519	(11.6)	7	(2.8)	< 0.0001	0.224
Continuous sedation	80	(1.8)	10	(4.1)	0.0108	2.335
Antibiotics and H_2 _antagonist	1,307	(29.1)	44	(17.9)	0.0001	0.529
Antibiotics and continuous sedation	319	(7.1)	31	(12.6)	0.0014	1.883
H_2 _antagonist and continuous sedation	183	(4.1)	15	(6.1)	0.1242	1.526
Antibiotics, H_2 _antagonist, and sedation	675	(15.1)	111	(45.1)	< 0.0001	4.640
No process of care	494	(11.0)	1	(0.4)	< 0.0001	0.033
Severity of illness, mean (SD)						
Baseline APACHE II score	19.4	(8.1)	23.5	(7.8)	< 0.0001	1.061
SOFA score indicator	6.6	(3.9)	9.0	(3.8)	< 0.0001	1.154
Laboratory data at baseline						
Serum creatinine > 2.0 mg/dl, number (percentage)	782	(17.6)	57	(23.2)	0.0250	1.417
Albumin ≤ 2.3 g/dl, number (percentage)	1,086	(29.8)	115	(54.0)	< 0.0001	2.763
Hemoglobin in g/dl, mean (SD)	10.8	(2.5)	10.3	(2.4)	0.0008	0.914
Transfusion						
Any transfusion, number (percentage)	1,892	(42.2)	164	(66.7)	< 0.0001	2.737
Units transfused, mean (SD)	1.8	(3.5)	3.8	(5.6)	< 0.0001	1.088

^a^These categories are not mutually exclusive. APACHE II, Acute Physiology and Chronic Health Evaluation II; ARDS, acute respiratory distress syndrome; ICU, intensive care unit; SD, standard deviation; SOFA, Sequential Organ Failure Assessment.

With respect to pRBC transfusion exposure, 2/3 (66.7%) of all ARDS cases had received a transfusion during the observation period compared to 42.2% (unadjusted OR, 2.74; 95% CI, 2.09 to 3.59) of the controls, suggesting an increasing incidence of ARDS as a function of transfusion status. Additionally, on average, patients developing ARDS received significantly more blood than the controls (3.8 versus 1.8 units per patient transfused, respectively; *p *< 0.0001), and there was a dose-response relationship observed between the amount of blood transfused and the risk of ARDS development such that unadjusted ORs relative to no transfusion were as follows: 1 to 2 units, 2.24 (95% CI, 1.60 to 3.14); 3 to 4 units, 2.33 (95% CI, 1.55 to 3.51); and more than 4 units, 3.89 (95% CI, 2.78 to 5.44) (*p *< 0.05 for all).

Table 2 presents the multivariate analysis of independent risk factors for ARDS. After duration of observation (that is, time at risk) was corrected for, the use of total parenteral nutrition, continuous sedation, early enteral feeding, and the admitting diagnosis of pneumonia were significantly associated with an increased risk of ARDS. Whereas severity of illness, baseline hemoglobin, and hypoalbuminemia were each significant risk factors for ARDS development, the magnitude of the association with ARDS and level of significance were less for each of these risk factors.

**Table 2:** Multivariate analysis of independent risk factors for acute respiratory distress syndrome

Variable	Adjusted odds ratio^a,b^	95% confidence limits	*P *value
Age of patient: ≥ 65 years old (reference: < 65 years old)	0.687	0.495–0.954	0.025
Admitting diagnosis (reference: no)			
Pneumonia	2.831	1.914–4.186	< 0.0001
Sepsis/Systemic inflammatory response syndrome	0.856	0.552–1.327	0.4875
Trauma	0.974	0.601–1.577	0.9133
ICU type			
Surgical ICU (reference: medical ICU and combined)	1.543	1.055–2.259	0.0255
Severity of illness			
SOFA score indicator (continuous variable)	1.078	1.034–1.124	0.0005
Process of care (reference: no)			
H_2 _antagonists at baseline	1.332	0.953–1.862	0.0931
Continuous sedation	4.237	3.000–5.983	< 0.0001
Nutritional status at baseline or ICU day 3–4 (reference: no)			
Total parental nutrition	4.659	3.064–7.082	< 0.0001
Enteral nutrition	3.153	2.189–4.540	< 0.0001
Laboratory data at baseline			
Albumin ≤ 2.3 mg/dl (reference: no)	1.930	1.390–2.680	< 0.0001
Hemoglobin level (continuous variable)	1.075	1.002–1.152	0.0435
Transfusion status			
Any transfusion (yes/no)	2.797	1.899–4.120	< 0.0001
Transfusion exposure (reference: no transfusion)^c^			
1–2 units transfused	2.191	1.409–3.407	0.0005
> 2 units transfused	3.784	2.417–5.924	< 0.0001

^a^All odds ratios were adjusted for duration of observation and other covariates. ^b^Other covariates not achieving the statistical significance entry criterion (*p *< 0.1) were gender; admitting diagnoses of neurological disorder, gastrointestinal disease, and chronic obstructive pulmonary disease; medical history of diabetes and malignancy; baseline APACHE II (Acute Physiology and Chronic Health Evaluation II) score; antibiotics use at baseline; total serum bilirubin of more than 2.0 mg/dl; and serum creatinine of more than 2.0 mg/dl. ^c^Estimated from a separate model in which the categorical transfusion variables replace the transfusion dichotomous variable. ICU, intensive care unit; SOFA, Sequential Organ Failure Assessment.

**Figure 1:**
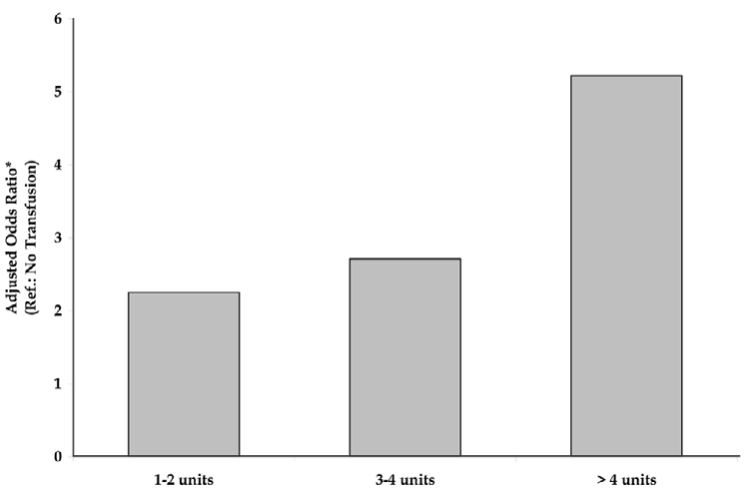
Multivariate analysis of independent transfusion risk factor for acute respiratory distress syndrome (ARDS). After covariates were adjusted for, the amount of blood exposure remained statistically significantly associated with an increasing risk of developing ARDS. Adjusted odds ratios relative to no transfusion were as follows: 1 to 2 units, 2.25 (95% confidence interval [CI], 1.44 to 3.50); 3 to 4 units, 2.71 (95% CI, 1.58 to 4.65); and more than 4 units, 5.22 (95% CI, 3.12 to 8.74) (*p *< 0.05 for all). *Covariates adjusted for in the multivariate logistic model included age, admitting diagnosis, intensive care unit type, nutritional status, process of care, severity of illness, and laboratory data.

Transfusion was independently associated with ARDS (Table 2). When transfusion was considered as a dichotomous variable (yes/no), there was a significant association between pRBC transfusion and ARDS development (adjusted OR, 2.80; 95% CI, 1.90 to 4.12; *p *< 0.0001). When examining the impact of the amount of pRBCs transfused, patients who received even small numbers of pRBCs (1 to 2 units) faced a more than two-fold increase in the risk for developing ARDS (adjusted OR, 2.19; 95% CI, 1.41 to 3.41; *p *= 0.0005) relative to patients receiving no transfusion. Transfusing larger amounts of blood (more than 2 units of pRBCs) further increased the risk for ARDS development to nearly four times that observed in patients not exposed to pRBC transfusion (adjusted OR, 3.78; 95% CI, 2.42 to 5.92; *p *< 0.0001). This dose-response relationship persisted even when transfusion exposure exceeded 4 units (Figure 1).

We further examined several hospital and ICU outcomes and compared them in the two groups (Table 3). The mean duration of mechanical ventilation was nearly triple in the ARDS group compared to the No ARDS group (*p *< 0.0001). Similarly, mean ICU and hospital lengths of stay were nearly three and two times as long, respectively, among patients with ARDS as among those without ARDS (*p *< 0.0001 for both). Mortality was also significantly higher in the group with ARDS when compared to the group without (hospital mortality: 37.8% and 16.1%, respectively, *p *< 0.0001; ICU mortality: 35.8% and 11.2%, respectively, *p *< 0.0001).

**Table 3:** Hospital and ICU outcomes during hospitalization period

Variable	No ARDS (*n *= 4,484)	ARDS (*n *= 246)	*P *value
Length of stay in days, mean (SD)			
Hospital length of stay	12.7 (8.7)	21.1 (9.3)	< 0.0001
ICU length of stay	6.7 (6.6)	17.7 (9.3)	< 0.0001
Mechanical ventilation			
On ventilator at ICU admission, number (percentage)	2,024 (45.1)	136 (55.3)	0.0019
Duration on ventilator in days, mean (SD)	6.2 (6.7)	15.4 (9.0)	< 0.0001
Mortality rate			
Hospital mortality, number (percentage)	721 (16.1)	93 (37.8)	< 0.0001
ICU mortality, number (percentage)	504 (11.2)	88 (35.8)	< 0.0001

ARDS, acute respiratory distress syndrome; ICU, intensive care unit; SD, standard deviation.

## Discussion

This secondary analysis of a prospective cohort of critically ill patients indicates that pRBC transfusion is associated with an increased risk of developing ARDS. This link is independent of multiple variables, including other important potential confounders such as severity of illness and reason for ICU admission. Furthermore, there appears to be a dose-response relationship between pRBC use and development of ARDS; receiving more pRBC units raises the likelihood of subsequent ARDS. In addition, progression to ARDS in the ICU substantially prolonged the duration of mechanical ventilation and was associated with a poor prognosis.

Three prior studies have focused expressly on ARDS as it relates to pRBC transfusion practice [8-10]. Hébert and colleagues [8] randomly assigned critically ill patients to withhold transfusions at or above a hemoglobin level of 7 versus 10 mg/dl. Despite receiving fewer units of pRBCs, those in the conservative hemoglobin arm (that is, 7 mg/dl) had similar overall survival rates compared to those in the comparator group. With respect to ARDS, 7.7% of those randomly assigned to the lower hemoglobin threshold developed ARDS versus 11.4% of those in the higher target cohort (*p *= 0.06). Kahn and colleagues [10] noted that pRBC transfusion independently more than doubled the probability of ARDS among a group of individuals suffering a subarachnoid hemorrhage. Gong and colleagues [9], in an analysis similar to ours, studied a mixed population of 688 critically ill patients at risk for ARDS. ARDS developed in approximately one third of the population. Adjusting for several covariates indicated that pRBC transfusion was significantly related to a diagnosis of ARDS and, importantly, was also associated with a greater mortality rate.

Our observations build on these earlier reports and confirm a relationship between pRBC exposure and ARDS. In contrast to Hébert and colleagues [8], Kahn and colleagues [10], and Gong and colleagues [9], we were able to control for multiple processes of care beyond transfusion practice alone. For example, we could adjust for use of continuous sedation. Likewise, we were able to consider severity of illness not only at ICU entry (APACHE II score), but also immediately prior to the onset of ARDS (SOFA score). This is important because as patients remain in the ICU their severity of illness changes. Failure to evaluate the evolution in disease severity might lead one to find a relationship where, in fact, the observation is simply a marker for alterations in severity of illness. Moreover, unlike the studies by Kahn and colleagues [10] and Gong and colleagues [9], ours is derived from a multi-institutional registry, and this underscores the likely generalizability of our data. An additional strength of our study stems from the large sample size. Having nearly 5,000 subjects to analyze afforded us statistical power to assess multiple covariates that other reports could not take into consideration.

Among other factors that we found were correlated with ARDS, several merit comment. Prior analyses of 'risk factors' for ARDS have not investigated either continuous sedation or early enteral feeding as potential process-of-care issues that could promote ARDS [2-5,9]. The impact of continuous sedation likely arises from the fact that this strategy (as compared to intermittent sedation protocols) prolongs the time on mechanical ventilation and hence the risk for ventilator-associated lung injury. On the other hand, since continuous sedation remained an independent predictor of ARDS after the period of observation was controlled for, it could be that this is simply a surrogate marker for severity of illness which is not captured by traditional scoring tools. The correlation between early enteral feeding could reflect the fact that this approach to nutrition heightens the probability of either gross aspiration or microaspiration. Both of these variables merit scrutiny in future analyses. As such, our work serves to generate hypotheses for future research.

Why might transfusion correlate with the development of ARDS? In fact, what is diagnosed as ARDS in the critically ill population may represent TRALI. Thus, the proposed mechanisms for TRALI – (a) alloimmunization of the recipient white cells by the donor anti-leukocyte [6] and monocyte antibodies [23] and (b) a response to biologically active lipids originating in donor plasma [24-27], resulting in an oxidative burst leading to degranulation of neutrophils after some priming event – could underlie the more general relationship we note between pRBC use and ARDS. Alternatively, several studies have shown that pRBCs contain multiple pro-inflammatory cytokines that, when infused into a susceptible subject, could potentially tip the balance and lead to a dysregulation of multiple cascades that have their clinical manifestation as acute lung injury [28-30]. Thus, transfusion promotes inflammation directly as demonstrated in studies that measure serial levels of interleukin-6 in the recipient following pRBC administration [12]. On the other hand, residual donor white blood cells could promote T-cell activation [29,30], which, in turn, could result in subtle changes in the host's immune status, predisposing him or her to infection. Whatever the mechanism, it may vary from individual to individual based on genetics and proteomics.

Our findings should give physicians pause when considering transfusion in persons at risk for ARDS. Evidence continues to mount that transfusion increases the risk for multiple adverse consequences ranging from bloodstream infections to nosocomial pneumonia [18,19,31-38]. A recent prospective study by Taylor and colleagues [32] explored the impact of pRBC use on subsequent rates of nosocomial infection. Although they pooled all types of infection into a common endpoint, they concluded that transfusion independently increases the risk for infection. In other analyses looking at distinct forms of nosocomial infection, such as pneumonia or bloodstream infection [18,19,33], researchers have reached similar conclusions regarding the potential deleterious effects of transfusion. All of these reports, including our own, are necessarily limited in that they can demonstrate only association rather than causation. However, given the consistent theme observed in multiple datasets, our results should help to shift the burden against assuming that pRBC exposure is free of substantial risk. Bolstering this recommendation to discard the assumption that transfusion is a relatively 'safe' endeavor is the fact that the relationship between pRBCs and ARDS in the present report follows a dose-response relationship. Even small transfusion volumes (for example, 1 to 2 units) convey an increased probability for the development of ARDS.

Our study has a number of significant limitations. First, although the data were collected prospectively, this report represents a retrospective analysis. As such, it is exposed to multiple forms of bias. Second, as our analysis describes observational data and does not derive from a randomized study, we can conclude only that transfusion is associated with the development of ARDS. Causation, therefore, cannot be inferred from our analytic approach. Third, the diagnosis of ARDS was based on a prospectively chosen definition, but these criteria were applied by a diverse group of researchers. Inter-observer variability in the diagnosis of ARDS [39,40], which has been documented in prior studies, could confound our findings. The large sample size, however, should limit the impact of this variability. Fourth, we lacked information on transfusions given prior to ICU admission. Thus, it is possible that we may have misclassified at least some of the exposure information. However, if present, this misclassification of exposure would be nondifferential and, if anything, would have resulted in a weaker association between transfusions and the development of ARDS than one that actually exists. In that same vein, we did not record information regarding the use of other forms of blood products. Fifth, there may be further variables we did not investigate or record that could have affected our findings. Finally, the Crit trial was conducted prior to the implementation of leukoreduction. Leukoreduction is thought to decrease the immunomodulatory effects of pRBC transfusion. Despite theoretical reasons to hypothesize that leukoreduction might prevent serious infectious and non-infectious complications in critically ill patients, clinical evidence of the benefit of leukoreduction is sparse [41-45]. Nonetheless (and notwithstanding these limitations), our observations are consistent with an emerging literature indicating that transfusion is not benign.

## Conclusion

In summary, pRBC use independently correlates with the development of ARDS in ICU patients at risk for this process. The link between transfusion administration follows a dose-response relationship, suggesting that exposure to any pRBC transfusion volume increases the probability for the onset of this severe complication. We urge clinicians to consider this information as they weigh the risks and benefits of transfusion in individual patients and to acknowledge that the burden of proof is shifting to suggest that transfusion avoidance may be the safer paradigm.

## Key messages

• Allogeneic red blood cell transfusion is an independent risk factor for the development of acute respiratory distress syndrome (ARDS) in the intensive care unit population.

• The association of allogeneic blood exposure and ARDS development follows a dose-response relationship.

• This information needs to be included in the clinician's risk-benefit analysis when faced with a transfusion decision for an individual critically ill patient.

## Abbreviations

APACHE II = Acute Physiology and Chronic Health Evaluation II; ARDS = acute respiratory distress syndrome; CI = confidence interval; ICU = intensive care unit; OR = odds ratio; pRBC = packed red blood cell; SIRS = systemic inflammatory response syndrome; SOFA = Sequential Organ Failure Assessment; TRALI = transfusion-related acute lung injury.

## Competing interests

At the time of this study and manuscript preparation, MDZ was an employee of Ortho Biotech Clinical Affairs, LLC (Bridgewater, NJ, USA). She currently serves as a consultant to Ortho Biotech Clinical Affairs, LLC, and is a stockholder in Johnson & Johnson (New Brunswick, NJ, USA), its parent company. CC is an employee of Ortho Biotech Clinical Affairs, LLC, and is a stockholder in Johnson & Johnson. At the time of this study and manuscript preparation, MR was an employee of Ortho Biotech Clinical Affairs, LLC, and is a stockholder in Johnson & Johnson. PL, FV, and MSD are employees of Analysis Group, Inc. (Boston, MA, USA), which has received research funds from Ortho Biotech Clinical Affairs, LLC. AFS is a consultant to and has received funding from Ortho Biotech Clinical Affairs, LLC.

## Authors' contributions

MDZ and AFS were responsible for the study design, data interpretation, and drafting the manuscript. CC and MR were responsible for the study design and data interpretation. PL, FV, and MSD were responsible for the study design, data analyses, and data interpretation. All authors read and approved the final manuscript.
